# Microstructure and Thermal Insulation Property of Silica Composite Aerogel

**DOI:** 10.3390/ma12060993

**Published:** 2019-03-26

**Authors:** Lei Shang, Yang Lyu, Wenbo Han

**Affiliations:** National Key Laboratory of Science and Technology on Advanced Composites in Special Environments, and Center for Composite Materials and Structures, Harbin Institute of Technology, Harbin 150080, China; shanglei601@163.com (L.S.); 15546125912@163.com (Y.L.)

**Keywords:** silica aerogel, anti-infrared radiation fiber, supercritical drying, thermal insulation

## Abstract

Tetraethyl orthosilicate was selected as a matrix of heat insulating materials among three silanes, and an anti-infrared radiation fiber was chosen as a reinforcement for silica aerogel insulation composite. The silica aerogel was combined well and evenly distributed in the anti-infrared radiation fiber. The heat insulation effect was improved with the increase in thickness of the aerogel insulation material, as determined by the self-made aerospace insulation material insulation performance test equipment. The 15 mm and 30 mm thick thermal insulation material heated at 250 °C for 3 h, the temperatures at the cold surface were about 80 °C and 60 °C, respectively, and the temperatures at 150 mm above the cold surface were less than 60 °C and 50 °C, respectively. The silica aerogel composites with various thicknesses showed good thermal insulation stability. The silica insulation composite with a thickness of 15 mm exhibited good heat insulation performance, meets the thermal insulation requirements of general equipment compartments under low-temperature and long-term environmental conditions. The thermal conductivity of prepared silica aerogel composite was 0.0191 W·m^−1^·k^−1^ at room temperature and 0.0489 W·m^−1^·k^−1^ at 500 °C.

## 1. Introduction

The design and manufacture of thermal protection structures are key technologies for high-temperature thermal protection of aircraft, particularly for the development of hypersonic vehicles [1,2,3]. When high-speed air bypasses the body, the air is strongly compressed and rubbed by the surface of the body. Most of the kinetic energy of the airflow is converted into heat energy, and so the temperature of the air rises rapidly. High-temperature air transfers a large amount of heat energy to the surface structure of the body, which greatly increases the temperature of the aircraft surface. For electronic and electrical equipment inside an aircraft body, the operating temperature should not exceed 85 °C. Such a high temperature difference will bring serious challenges to aircraft thermal protection. To ensure the normal operation of the aircraft’s internal equipment, it needs to be thermally protected by a high-performance a thermal protection structure [4,5,6,7]. European and American countries, particularly the United States, have been researching thermal protection systems for more than half a century, forming a comprehensive system with mature technologies. Its structure includes semi-active thermal protection, active thermal protection, and mainly passive thermal protection. Part of the high-Mach-number verification machine uses a semi-active thermal protection structure with relatively mature technology. Active thermal protection structures are mainly in the research phase and have not entered the engineering application phase. China’s thermal protection structure research started late, with fewer technical reserves, and the structural forms are passive protection systems. Domestic aircraft in service have a clear and urgent need for thermal protection materials and structural design. The service environment of the aircraft is deteriorating especially with the development of high-vibration speed aircraft. The existing thermal protection structure design guidelines and insulation materials can no longer meet the model design requirements. Thermal protection technology has become a bottleneck that hinders the development of hypersonic vehicles.

Given the current situation and development needs, silica aerogel materials have shown a special structure of low density, high porosity, and high specific surface area. The heat-resistant, heat-insulating, and mechanical properties of functional composites with silica aerogel (matrix) and fiber (reinforcement) are mainly determined by the microstructure of the aerogel and the structure and composition of the fiber preform. However, the thermal insulation of these fiber-strengthened silica aerogels was compromised. However, aerogels have high brittleness, low strength, and infrared transparency and cannot be directly applied to engineering. Aerogels must be reinforced with an anti-infrared radiation fiber preform. By introducing the anti-infrared radiation fiber preform and controlling the structure and composition of the fiber preform, the mechanical properties and thermal insulation properties of the aerogel insulation material can be improved. Liu et al. prepared a silica aerogel with a thermal conductivity of 0.0486 W·m^−1^·k^−1^ [8]. And, the thermal insulation of these silica aerogels was affected by fiber-reinforced. For example, the thermal conductivity of a silica aerogel reinforced by aramid, mineral, ceramic fibers was increased to a corresponding value of 0.0232 W·m^−1^·k^−1^ [9], 0.025 W·m^−1^·k^−1^ [10], 0.041–0.084 W·m^−1^·k^−1^ [11,12,13]. Moreover, White et al. prepared aerogel materials in a few hours using supercritical drying methods [14]. And Saeed et al. using laser-induced gelation method, which can significantly improve the degree of crosslinking and further decreased the processing time of aerogel materials [15].

**Figure 1:**
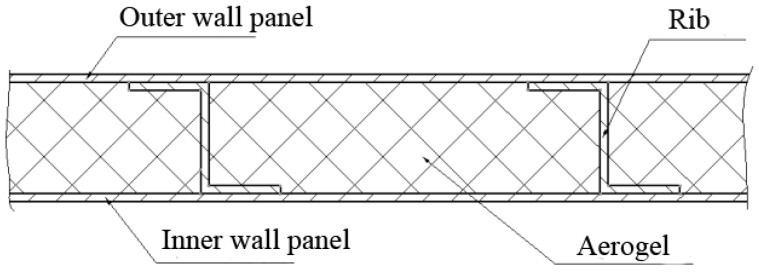
Large insulation material structure design.

To produce large protective materials for insulation, the typical structure includes a single-layer skin-insulation material, reinforced wall board-insulation material, inner and outer skin insulation material, inner and outer skin-rib-heat insulation material, honeycomb structure-insulation materials, and other structural forms. The structural design of large thermal insulation materials is shown in Figure 1. Structural materials are, respectively, selected from titanium alloys, titanium–aluminum, and high-temperature alloys, which have excellent heat resistance. The aerogel is added in the middle of the wall or in the middle of the inner and outer wall panels, and the thickness ranges from 2 mm to 35 mm to form several groups of structures. In this paper, the structure, preparation, and properties of a double-layer skin-rib (20 mm high)-aerogel (adhesive) were investigated. Compared with the thermal conductivity of other kinds of fiber reinforced silica aerogels, the anti-infrared radiation fibers reinforced silica aerogel composite is lower. In general, the total thermal conductivity of fiber-reinforced aerogel composites is the sum of the three components of solid heat transfer, gas heat transfer, and radiation heat transfer [16]. The anti-infrared radiation fiber has a strong anti-radiation effect and low thermal conductivity. Thus, the anti-infrared radiation fibers reduce the radiant heat transfer of the fiber reinforced silica aerogel composite.

## 2. Experimental Procedure

### 2.1. Material Preparation

**Figure 2:**
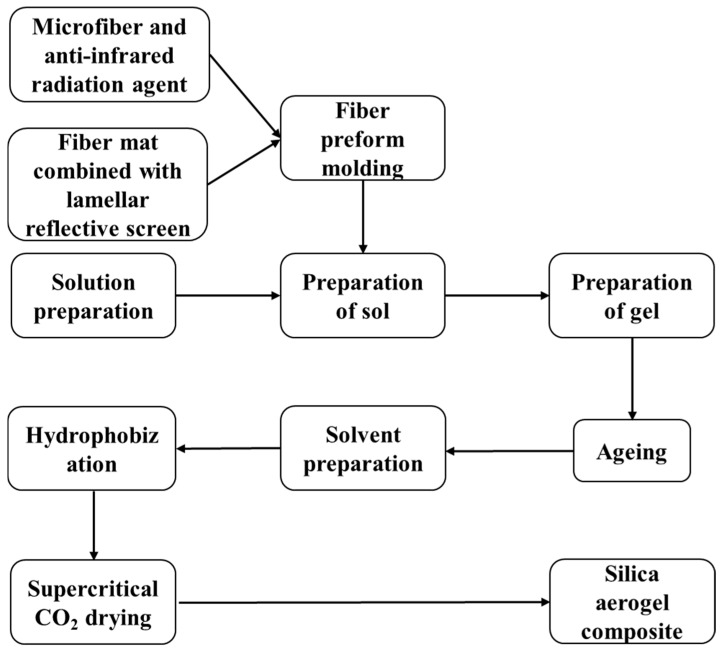
Silica aerogel composite development process.

Anti-infrared radiation silica fibers raw material (Guangwei Composite Materials Co., Ltd., Weihai, China) were subjected to acid filtration in a 5 to 6% mass fraction of hydrochloric acid at a temperature of 90 to 95 °C for 100 min to obtain the anti-infrared radiation silica fibers having a silica content more than 91% with a little MgO and CaO. Ethyl orthosilicate (TEOS), methyl orthosilicate (TMOS) and polysiloxane (Si40) (Aladdin Holdings Group Co., Ltd., Shanghai, China) were used as silanes for the preparation of silica aerogels. The synthesis of anti-infrared radiation silica fiber reinforced silica aerogel composites with different silanes is shown in Figure 2. In this work, the main preparation process of the silica aerogel composite insulation material can be described as follows: A silane was used as a starting material. Through the sol-gel process, the prepared sol was first impregnated with a fiber preform, and then gelled to obtain a fiber-reinforced wet gel. During the post-treatment technique (i.e., aging, solvent exchange, and hydrophobization [17]), the wet gel samples were heated and immersed using a mixed reaction solution of tetraethyl orthosilicate and ethanol (molar ratio of 5:95). The immersed time was about 7 days. The reaction solution penetrates through diffusion, causing the unreacted groups to continue the polycondensation reaction, forming a new crosslinking unit in the original network, further completing the nanostructure of the gel and excluding water in the wet gel to obtain an alcogel, thereby, increasing the degree of crosslinking of the network structure. Finally, the aerogel insulation material was prepared by removing the ethanol in the alcogel exchange by a supercritical (SC) CO_2_ drying technique, with a drying temperature of 31.0 °C and drying pressure of 7.29 MPa. The preparation process was without any catalyst and other additives.

### 2.2. Measurement and Characterization

As the sample may be deformed at high temperatures, it is difficult to ensure good contact between the sample and the hot plate, and the external pressure clamping system will destroy the normal heat flow state inside the sample. Therefore, the hot-air test is the most ideal test method for high test temperature and good contact between the sample and the heat source [18,19,20].

In spacecraft thermal protection design verification, several radiation and airflow heat transfer modes were used. With reference to relevant domestic and foreign experimental equipment and devices, on the basis of the existing hot-air insulation performance testing equipment, the key issues of cold-surface insulation state simulation, heat loss and heat diffusion, temperature control accuracy, test accuracy, and test repeatability were solved. A set of thermal insulation performance testing equipment was built for standard samples of aviation thermal insulation materials.

**Figure 3:**
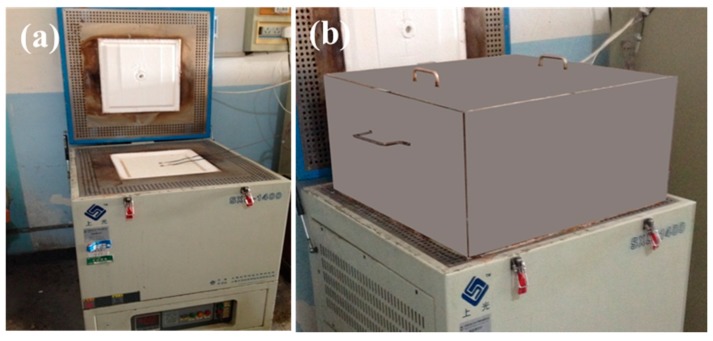
The upper open-type test electric furnace. (**a**) Upper opening heating furnace and (**b**) insulated enclosure.

The upper open-type test electric furnace was established, as shown in Figure 3a. The long-term working temperature was not lower than 1400 °C, the temperature uniformity at the furnace mouth should not be higher than 1% of the test temperature, and the temperature fluctuation at the furnace mouth should not be higher than 0.5% of the test temperature. An insulated enclosure for simulating a cold-faced enclosed environment was designed, as shown in Figure 3b. The top cover of the cover box had a temperature sensor jack, and a square port was left at the bottom, corresponding to the test furnace mouth. A low thermal insulation material with a thickness of 8 mm to 10 mm was placed on the inner side and the outer side of the cover. Specific surface area and pore width of silica aerogel prepared from different silanes by BET test method (BSD-PM1, Beishide, Beijing, China). The morphologies of the fibers and silica composite aerogel insulation materials were investigated by scanning electron microscopy (SEM, Quanta 200FEG, New York, NY, USA), operating with an accelerating voltage of 20 kV. Transmission electron microscopy (TEM, JEOL 2100, Tokyo, Japan) was used for the morphological characterization of the samples. The compressive strength of the silica composite aerogel insulation was measured according to Universal testing machine (AG-X Plus 20 kN/5 kN, Tokyo, Japan) operating strain rate with 2 mm/min. Solid-state nuclear magnetic resonance (NMR) spectra were acquired on a Bruker DRX300WB spectrometer (CPMAS-TOSS, Shanghai, China). The measurement time was sample dependent because the number of scans ranges from 1000 to 2000 for 29Si.

The density of the silica composite aerogel insulation was measured according to the ASTM (American Society of Testing Materials) C201-93 standard. The thermal conductivity meter of the TC 3000E model was adopted. The measuring principle of the device was the transient hot wire method, and the thermal conductivity of the measured medium was calculated.

## 3. Result and Discussion

### 3.1. Microstructure Characterization

**Figure 4:**
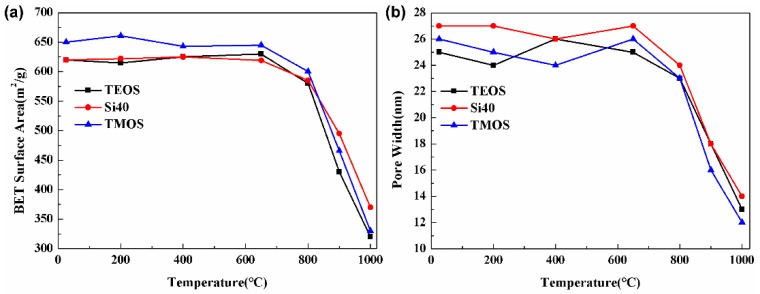
Specific surface area and pore width of silica aerogels prepared by three different silanes systems varied with temperatures: (**a**) specific surface area and (**b**) pore width.

Specific surface area and pore size are two important indicators that affect the thermal conductivity and thermal insulation of aerogels. TMOS, TEOS, and Si40 were used to prepare aerogels. The changes of specific surface area and average pore size after treatment at different treatment temperatures for 30 min are shown in Figure 4. In Figure 4a, the aerogels prepared by using the three silanes have a specific surface area of more than 600 m^2^/g at room temperature. The aerogels prepared using the three silanes were heated to 600 °C. The specific surface area did not change significantly, indicating that the microstructure was highly stable. When the heating temperature exceeds 800 °C, the specific surface area rapidly decreased, indicating that the microstructure began to change significantly. The pore size of the aerogel did not change significantly after treatment at 600 °C. When the treatment temperature was increased to 800 °C, the pore size of the aerogel rapidly decreased, as shown in Figure 4b. This observation indicated that the nanoparticles were sintered, resulting in pore structure to collapse. The microstructure of the silica aerogel prepared from the organic silane determines the difference in temperature resistance. In a high-temperature environment, the nanoparticles in the aerogel structure would sinter and aggregate, causing the “collapse” of the nanopore structure, and, thus, failure of the three-dimensional network structure of the material. As shown in Figure 4b, the aerogel prepared by TEOS at around 200 °C has the lowest specific surface area and the smallest pore size. Moreover, the structure was moderately controllable at a moderate temperature. Compared with TMOS and Si40, TEOS was an ideal silane for the preparation of medium and low-temperature aerogel insulation materials.

**Figure 5:**
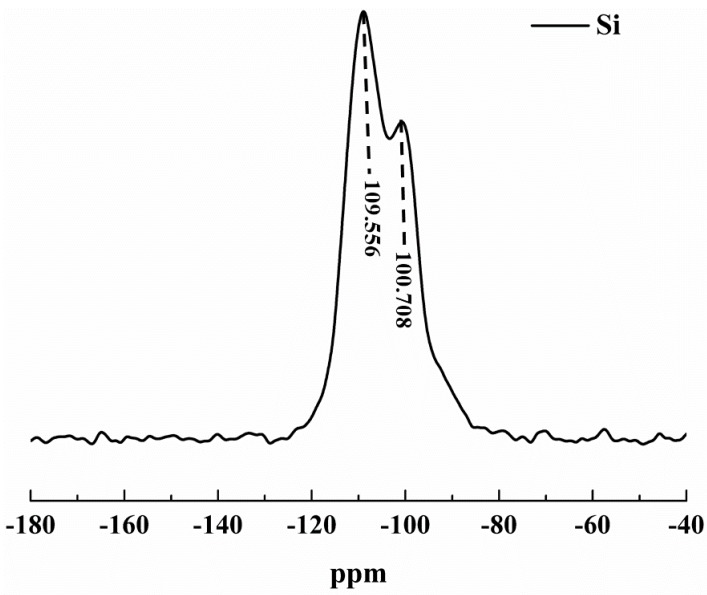
Solid-state nuclear magnetic resonance (NMR) 29Si spectrum for supercritical (SC) CO_2_ drying cross-linked aerogels.

Solid-state nuclear magnetic resonance (NMR) analysis was performed on the samples to further confirm the formation of the network structure and to investigate any anomalies due to alcoholysis, hydrophobization or supercritical (SC) CO_2_ drying during the preparation of the aerogel. The spectrum obtained with 29Si is shown in Figure 5. The 29Si spectra of cross-linked samples containing silica networks (Figure 5) show two major characteristic peaks, one due to the presence of silane at −100.708 ppm and the other due to siloxane structure at −109.556 ppm. The peaks in other areas are baseline noise. No peak at −52 ppm and −59 ppm confirmed that TEOS had no single bond or double bond with the silica skeleton, which further confirmed the completion of the alcoholysis and condensation reactions. Because the samples were analyzed using supercritical (SC) CO_2_ drying, no peak representing silicon atoms was found at −93 ppm [21]. This confirms that all the prepared samples have completed the alcoholysis and condensation reactions, thus, forming the silica network structure.

**Figure 6:**
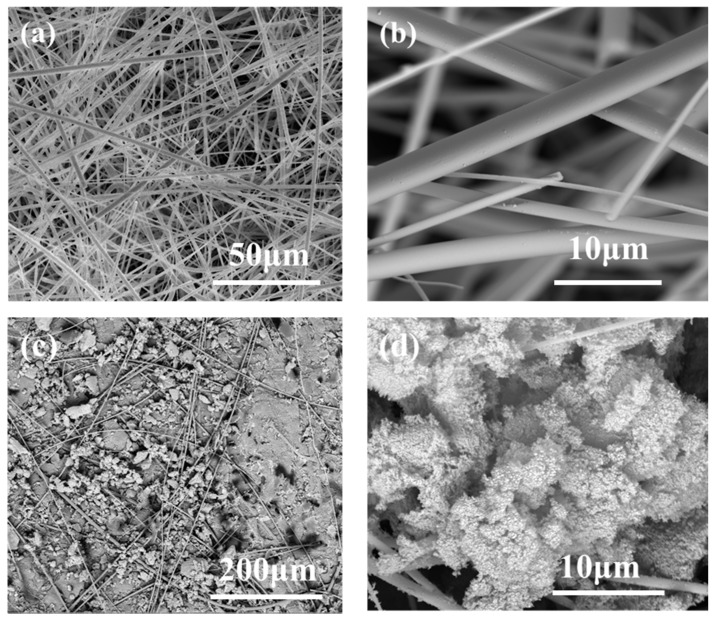
Microstructure of fiber (**a**) 50 μm; (**b**) 10 μm. And microstructure of aerogel insulation materials at different scales: (**c**) 200 μm; (**d**) 10 μm.

Figure 6 shows an SEM image of the composite obtained by impregnating the silanes into the anti-infrared fiber preform. Figure 6a,b is microscopic images of uncomposited anti-infrared radiation silica fibers. No agglomeration and entanglement were found between the anti-infrared radiation silica fibers, which were evenly distributed, and the anti-infrared radiation silica fibers had a smooth surface with a diameter of about 1–3 μm. In Figure 6c,d, the microscopic images of aerogel composites, which have smaller aerogel size and higher specific surface area. Moreover, the distribution in the fiber matrix was relatively uniform, and a good combination was obtained between the aerogel and the fiber. The difference in shrinkage between the fiber reinforcement and the aerogel matrix was reduced, thereby, decreasing the residual stress in the aerogel and ensuring the low thermal conductivity of the fiber-reinforced aerogel composite. Thus, the reaction system, post-treatment technology, and multi-component silane types had an influence on the aerogel microstructure. In summary, the composite maintains the aerogel properties in the microstructure. The pore size was much smaller than the mean free path of the air molecules in the atmosphere (70 nm), which greatly reduces the gas phase heat transfer and helps to reduce the heat convection [22].

**Figure 7:**
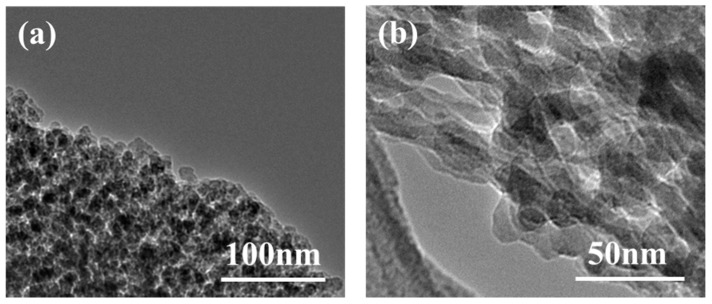
Transmission electron microscopy (TEM) micrographs of SiO_2_ aerogel: (**a**) 100 nm; (**b**) 50 nm.

The TEM micrographs, shown in Figure 7, clearly show the details of the internal aerogel structure. TEM image (a) confirmed particles were coated with ethyl alcohol. TEM image (b) further confirmed the interconnected structure and showed that the skeleton structure consisted of particles with a diameter ranging from 20 to 30 nm.

### 3.2. Thermal Insulation Performance of Aerogel

The silica composite aerogel insulation material density was measured according to ASTMC201-93 standard. The bulk density was as low as 0.144 g/cm^3^. Moreover, the thermal conductivity of the prepared anti-infrared radiation silica fibers reinforced silica aerogel composite was 0.0191 W·m^−1^·k^−1^ at room temperature and 0.0489 W·m^−1^·k^−1^ at 500 °C by thermal conductivity meter with TC 3000E model.

**Figure 8:**
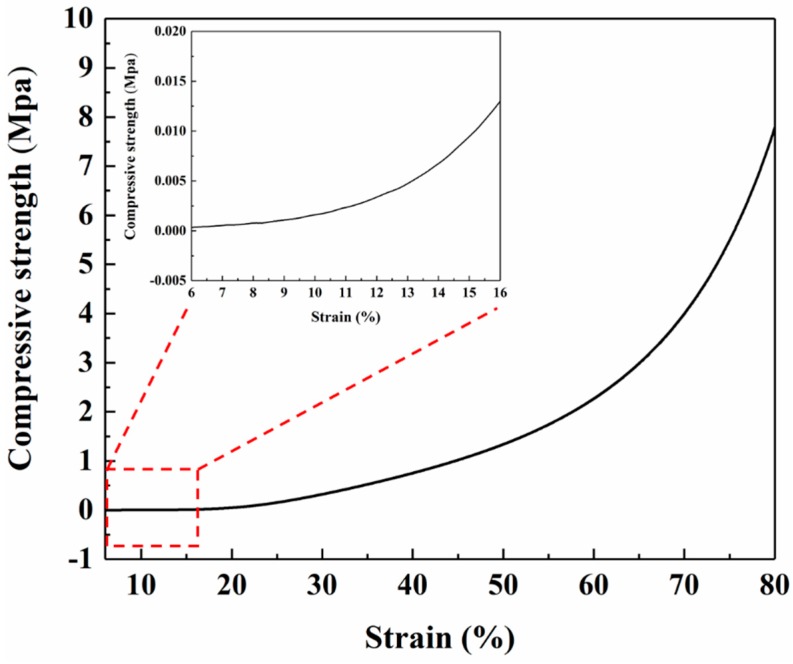
Compressive strength (MPa) of aerogel insulation materials (10 mm × 10 mm × 10 mm) with different strains and partial enlarged detail.

The relationship between compressive strength and strain was obtained by compressive mechanics experiments, as shown in Figure 8. It can be seen from the curve that the numerical change of compressive strength mainly occurs after 6% strain. When the strain was 25%, the rate of increase of compressive strength was obviously large. When the strain was 80%, the compressive strength reached a maximum of 7.71 MPa.

**Figure 9:**
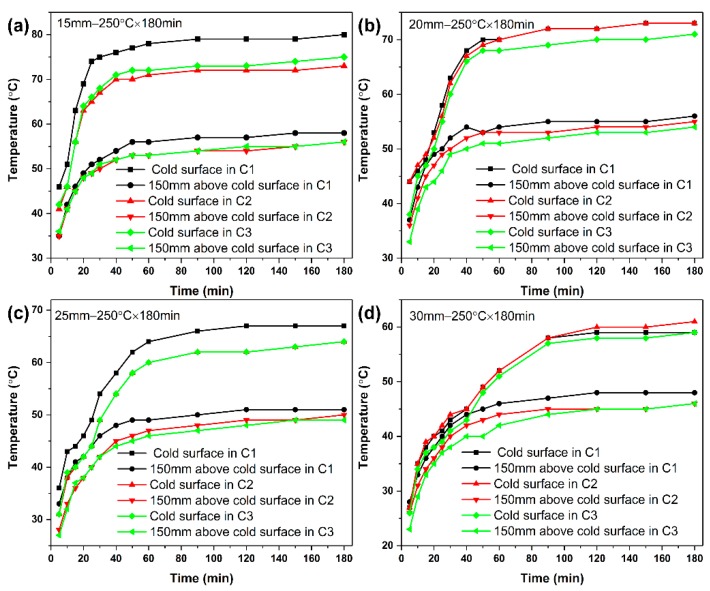
Temperatures on cold surfaces and 150 mm above the cold surfaces of aerogel insulation materials with different thickness during 3 thermal cycles: (**a**) 15 mm; (**b**) 20 mm; (**c**) 25 mm; (**d**) 30 mm.

The insulation performance of aerogel insulation materials (TEOS as silane) with various thicknesses (15 mm, 20 mm, 25 mm, and 30 mm) were investigated. The aerogel insulation material has a hot surface temperature of 250 °C and an ambient temperature of 25 to 27 °C. The thermal insulation effect was evaluated by measuring the temperature at the cold surface and 150 mm above the cold surface of the aerogel insulation composite. The temperature variation curves of the aerogel insulation materials with different thicknesses are shown in Figure 9. As shown in Figure 9a, the temperatures of the 15 mm aerogel insulation material at 150 mm above the cold surface and the cold surface after heating for 60 min tended to be stable. The steady-state temperatures at 150 mm above the cold and cold faces in the first thermal cycle reached 57 °C and 78 °C, respectively. The steady-state temperatures above the cold and cold surfaces tested in the second and third thermal cycles were both reduced, and the temperature difference between the two thermal cycles was small, with steady-state temperatures of approximately 55 °C and 73 °C, respectively. As shown in Figure 9b, the temperatures of the 20 mm aerogel insulation material at 150 mm above the cold surface and the cold surface after heating for 80 min tended to be stable. The steady-state temperatures at 150 mm above the cold and cold surface in the first thermal cycle reached 55 °C and 72 °C, respectively. The cold surface temperature in the second thermal cycle was substantially the same as that in the first thermal cycle. The temperature in the third thermal cycle decreased compared to the previous two cycles. The temperatures of the 25 mm aerogel insulation material at 150 mm above the cold surface and the cold surface after heating for 100 min tended to be stable. The steady-state temperatures at 150 mm above the cold and cold surface in the first thermal cycle reached 51 °C and 67 °C, respectively, which were lower than the steady-state temperatures of 15 mm and 20 mm insulation, as shown in Figure 9c. The cold surface temperature in the second thermal cycle was basically the same as that in the third thermal cycle. The steady temperature at 150 mm above the cold surface was also relatively close, both below 50 °C. When the thickness of the insulation material was increased to 30 mm, the aerogel insulation effect was better, as shown in Figure 9d. The temperatures of the 30 mm aerogel insulation material at 150 mm above the cold surface and the cold surface after heating for 120 min tended to be lower. The steady-state temperature of the cold surface was around 60 °C, and the steady-state temperature at 150 mm above the cold surface was about 48 °C, which was lower than the steady-state temperature of the cold surface of 15 mm and 20 mm insulation and 150 mm above the cold surface. The heating cycle was continued twice, and the temperature of the tested aerogel insulation was lower at different temperatures. The steady-state temperature of the cold surface was lower than 60 °C, and the steady-state temperature at 150 mm above the cold surface was about 45 °C. The large particles of silica gel and the nanopores of the silica aerogel serve as the main part [22], while the fibers integrate the glass fiber/aerogel composite at this stage. Silica aerogel contains micropores with good thermal insulation properties and its own low thermal conductivity. And the anti-infrared radiation fiber has a strong anti-radiation effect and low thermal conductivity [16,23]. Therefore, the anti-infrared radiation fiber reinforced silica aerogel composite has excellent thermal insulation properties, and the cold surface has a lower temperature. The steady-state temperature tables of samples with different cycles and thicknesses are shown in Table 1.

The above data showed that the heat insulation effect increased with the increase in thickness of the aerogel insulation material. The aerogel insulation performance of each specification was reproducible, and the curve was highly consistent. After repeated use, the thermal insulation performance did not decrease. Therefore, the aerogel insulation material prepared in this study was stable over a long time at a high-temperature oxygen environment of 250 °C. The aerogel composite material also had excellent heat insulation performance and was suitable for long-term use in a high-temperature environment of 250 °C. Under low-temperature and long-term environmental conditions, the aerogel insulation material had good heat insulation effect. The 15 mm insulation material can meet the temperature control requirements of general equipment compartment, and the thicker insulation material shows better insulation effect.

## 4. Conclusions

The aerogel was evenly distributed in the anti-infrared radiation fiber matrix, and a good combination between the aerogel and the fiber was achieved. The thermal conductivity of prepared silica aerogel composite was 0.0191 W·m^−1^·k^−1^ at room temperature and 0.0489 W·m^−1^·k^−1^ at 500 °C. The heat source of a 15 mm-thick heat-insulating material with a surface temperature of 250 °C is heated for 3 h, and the temperature at 150 mm above the cold surface and the cold surface did not exceed 60 °C and 80 °C, respectively. The steady-state temperature of the cold surface of 30 mm-thick aerogel insulation was about 60 °C, and the steady-state temperature of 150 mm above the cold surface was lower than 50 °C. The aerogel insulation performance of each specification was reproducible, and the curve was highly consistent. The 15 mm insulation material can meet the temperature control requirements of general equipment cabins under low-temperature and long-term environmental conditions.

## Figures and Tables

**Table 1:** Steady-state temperature table for samples at different cycles and thicknesses. (C_1_, _2_, _3_ represent the temperature of the different thermal cycles of the cold surface, C_1_, _2_, _3_-150 represent the temperature of different thermal cycles 150 mm above the cold surface.).

	C_1_	C_1_-150	C_2_	C_2_-150	C_3_	C_3_-150
15 mm, 60 min	78 °C	56 °C	71 °C	53 °C	72 °C	53 °C
20 mm, 80 min	72 °C	55 °C	72 °C	53 °C	69 °C	52 °C
25 mm, 90 min	66 °C	50 °C	62 °C	48 °C	62 °C	47 °C
30 mm, 120 min	58 °C	47 °C	58 °C	45 °C	57 °C	44 °C

## References

[1] Bolender M.A., Doman D.B. (2012). Nonlinear Longitudinal Dynamical Model of an Air-Breathing Hypersonic Vehicle. J. Spacecr. Rockets.

[2] Parker J.T., Serrani A., Yurkovich S., Bolender M.A., Doman D.B. (2007). Control-Oriented Modeling of an Air-Breathing Hypersonic Vehicle. J. Guid. Control Dyn..

[3] Su X., Jia Y., Du J. (2015). Modeling and input-output decoupling of hypersonic vehicles. Int. J. Control Autom. Syst..

[4] Savino R., Fumo M.D.S., Paterna D., Serpico M. (2005). Aerothermodynamic study of UHTC-based thermal protection systems. Aerosp. Sci. Technol..

[5] Glass D.E. Ceramic Matrix Composite (CMC) Thermal Protection Systems (TPS) and Hot Structures for Hypersonic Vehicles. Proceedings of the 15th AIAA International Space Planes and Hypersonic Systems and Technologies Conference (AIAA).

[6] Zhou X.F., Xiao H.N., Jian F. (2012). Preparation, properties and thermal control applications of silica aerogel infiltrated with solid–liquid phase change materials. J. Exp. Nanosci..

[7] Liu D.H. (2011). Heat Transfer Characteristics of High Temperature Multilayer Thermal Insulations. Aerosp. Mater. Technol..

[8] Liu Z.H., Ding Y.D., Wang F., Deng Z.P. (2016). Thermal insulation material based on SiO_2_ aerogel. Constr. Build. Mater..

[9] Li Z., Gong L., Cheng X., He S., Li C., Zhang H., Zhi L. (2016). Flexible silica aerogel composites strengthened with aramid fibers and their thermal behavior. Mater. Des..

[10] Li X., Wang Q., Li H., Ji H., Sun X., He J. (2013). Effect of sepiolite fiber on the structure and properties of the sepiolite/silica aerogel composite. J. Sol-Gel Sci. Technol..

[11] Hong C.Q., Han J.C., Zhang X.H., Du J.C. (2013). Novel nanoporous silica aerogel impregnated highly porous ceramics with low thermal conductivity and enhanced mechanical properties. Scr. Mater..

[12] Shi D., Sun Y., Feng J., Yang X., Han S., Mi C., Jiang Y., Qi H. (2013). Experimental investigation on high temperature anisotropic compression properties of ceramic-fiber-reinforced SiO_2_ aerogel. Mater. Sci. Eng. A.

[13] Li S., Wang C.-A., Hu L., Wang C. (2013). Improved Heat Insulation and Mechanical Properties of Highly Porous YSZ Ceramics After Silica Aerogels Impregnation. J. Am. Ceram. Soc..

[14] White L.S., Bertino M.F., Saeed S. (2015). Influence of silica derivatizer and monomer functionality and concentration on the mechanical properties of rapid synthesis cross-linked aerogels. Microporous Mesoporous Mater..

[15] Saeed S., Al Soubaihi R.M., White L.S., Bertino M.F., Saoud K.M. (2016). Rapid fabrication of cross-linked silica aerogel by laser induced gelation. Microporous Mesoporous Mater..

[16] Lei Y., Chen X., Hu Z., Cao B. (2017). A general strategy for improving the thermal insulation performance of aerogels by multiple impregnation. Scr. Mater..

[17] Fan H., Peng Y., Li Z., Chen P., Jiang Q., Wang S. (2013). Preparation and characterization of hydrophobic PVDF membranes by vapor-induced phase separation and application in vacuum membrane distillation. J. Polym. Res..

[18] Hu H.K., Gan L.H., Li G.G. (2000). Supercritical Drying Technology. Lab. Res. Explor..

[19] Wang Q., Li G., Zhao B., Zhou R.X. (2010). Synthesis of La Modified Ceria-Zirconia Solid Solution by Advanced Supercritical Ethanol Drying Technology and its Application in Pd-only Three-Way high silica fibers. Appl. Catal. B Environ..

[20] Wang B. (2004). Brief introduction of ASTM standard on physical testing. Phys. Test. Chem. Anal. Part A Phys. Test..

[21] Hanus M., Kabeláč M., Rejnek J., Ryjáček F., Hobza P. (2004). Correlated ab Initio Study of Nucleic Acid Bases and Their Tautomers in the Gas Phase, in a Microhydrated Environment, and in Aqueous Solution. Part 3. Adenine. J. Phys. Chem. B.

[22] Li C., Cheng X., Li Z., Pan Y., Huang Y., Gong L. (2017). Mechanical, thermal and flammability properties of glass fiber film/silica aerogel composites. J. Non-Cryst. Solids.

[23] Li Z., Cheng X., He S., Zhi L., Shi X.J., Gong L.L., Zhang H.P. (2016). Aramid fibers reinforced silica aerogel composites with low thermal conductivity and improved mechanical performance. Compos. Part A Appl. Sci. Manuf..

